# A REFLECTION OF A STUDENT-LED VIRTUAL REALITY ADOPTION PROGRAM FOR PEOPLE LIVING WITH DEMENTIA IN LONG-TERM CARE

**DOI:** 10.1093/geroni/igae098.3066

**Published:** 2024-12-31

**Authors:** Joey Wong, Karen Lok Yi Wong, Mary Van, Yumna Hayat, Christine Wallsworth, Jim Mann, Lily Wong, Lillian Hung

**Affiliations:** University of British Columbia, Vancouver, British Columbia, Canada; University of British Columbia, Vancouver, British Columbia, Canada; University of British Columbia, Vancouver, British Columbia, Canada; University of British Columbia, Vancouver, British Columbia, Canada; University of British Columbia, Vancouver, British Columbia, Canada; University of British Columbia, Vancouver, British Columbia, Canada; University of British Columbia, Vancouver, British Columbia, Canada; University of British Columbia, Vancouver, British Columbia, Canada

## Abstract

Previous research suggests that virtual reality (VR) holds promise in improving residents’ well-being in long-term care (LTC). With ongoing staff shortages, there is a critical need to explore, evaluate and share learnings on innovative approaches for adopting technologies, including VR, in LTC. In our research study conducted at two LTC homes in Vancouver, Canada, undergraduate students supported staff members in VR adoption. This study reflects on the opportunities and challenges of VR adoption from the students’ perspectives. Utilizing a critical reflection framework, our interdisciplinary team engaged in extensive discussions and reflections via Zoom meetings, resulting in the following five themes: (1) Challenging the stigma towards dementia in LTC, (2) Addressing ageism in older adults’ technology usage, (3) Building relationships through consistent and repeated visits to facilitate technology adoption, (4) Recognizing students’ value in technology implementation and their potential impact on LTC’s future workforce, and (5) Recognizing the importance of emotional and organizational support. Based on these reflections, our team devised the “ENRICH” framework, offering six practical tips to support student involvement in enhancing technology adoption in LTC. The team’s insights highlight the potential of engaging university students in promoting technology adoption in LTC settings. The “ENRICH” framework provides a practical approach to fostering technology uptake while offering valuable learning experiences for students. This study encourages further exploration of student participation in LTC research to advance future initiatives in this field.

